# Safety of Levodopa-Carbidopa Intestinal Gel Treatment in Patients with Advanced Parkinson's Disease Receiving ≥2000 mg Daily Dose of Levodopa

**DOI:** 10.1155/2020/9716317

**Published:** 2020-02-13

**Authors:** Cindy Zadikoff, Werner Poewe, James T. Boyd, Lars Bergmann, Horia Ijacu, Pavnit Kukreja, Weining Z. Robieson, Janet Benesh, Angelo Antonini

**Affiliations:** ^1^AbbVie Inc., North Chicago, Illinois, USA; ^2^Department of Neurology, Medical University of Innsbruck, Innsbruck, Austria; ^3^Department of Neurology, University of Vermont College of Medicine, Burlington, Vermont, USA; ^4^Department of Neurosciences, Padua University, Padua, Italy

## Abstract

**Background:**

Levodopa-carbidopa intestinal gel (LCIG) provides continuous levodopa administration and clinical benefits to patients with advanced Parkinson's disease (PD). This report evaluates long-term safety and efficacy of high-dose LCIG in PD patients.

**Methods:**

Data were collected from several prospective, phase III clinical studies and an observational registry. The phase III program (*N* = 412) included four multicenter studies: a 12-week, randomized, double-blind study and three open-label studies extending ≥12 months. GLORIA (*N* = 412) included four multicenter studies: a 12-week, randomized, double-blind study and three open-label studies extending ≥12 months. GLORIA (

**Results:**

A total of 72 of 412 (17.5%) patients required dosages ≥2000 mg/day LCIG in the phase III program and 47 of 375 (12.5%) patients in GLORIA. Baseline demographics and disease severity were similar between dosage groups with more men in the high-dosage group. Compared with the <2000 mg/day dosage group, patients requiring ≥2000 mg/day LCIG had higher rates of AEs/ADRs including polyneuropathy; improvements in “Off” time and discontinuations due to AEs were similar between dosage groups and lower for discontinuations due to ADRs reported in GLORIA.

**Conclusions:**

Patients who require ≥2000 mg/day LCIG exhibited a safety profile comparable to the established safety/tolerability of LCIG with similar clinical improvements. Higher AEs were noted but within what is accepted for LCIG. Continuous administration of LCIG is beneficial to advanced PD patients who require very high doses of levodopa.

## 1. Introduction

Patients with Parkinson's disease (PD) treated with levodopa may develop long-term complications of therapy, including motor fluctuations in “On”/“Off” time and debilitating dyskinesias disrupting quality of life [1]. Longitudinal management of motor complications is typically approached with dose escalation and fragmentation of levodopa as well as adjunct therapies such as monoamine oxidase B (MAO-B) or catechol-O-methyl transferase (COMT) inhibitors or dopamine agonists [2, 3]. These long-term complications are attributed in part to the natural progression of the disease; the short half-life of levodopa; and the unstable, noncontinuous dosing regimens that result in pulsatile stimulation of dopamine receptors [4–6]. Accordingly, pharmacological delivery systems that provide more consistent dopamine replacement have been of interest.

Delivery routes that provide more continuous administration of levodopa (e.g., intestinal) show increased stability in drug plasma levels when compared with orally administered levodopa and improved symptomatic control in patients with advanced PD [7]. Levodopa-carbidopa intestinal gel (LCIG, also known as carbidopa-levodopa enteral suspension in the United States) was developed to deliver continuous administration of levodopa treatment via percutaneous endoscopic gastrojejunostomy (PEG-J), resulting in improved motor function and fewer drug fluctuations in patients with advanced PD. Results from subsequent clinical studies using this approach have demonstrated improvements in clinical outcomes, including significant improvements in “Off ” time and “On” time without troublesome dyskinesia (TSD), quality of life, and improvements in activities of daily living [8–12]. Although there is robust evidence for the clinical benefit of LCIG in patients with advanced PD, there are no studies assessing the safety and efficacy of high doses (≥2000 mg), and information on the use of high-dose LCIG is limited. Additionally, in the United States, the maximum recommended dose of levodopa is 2000 mg.

In this report, we describe safety and efficacy findings from a combination of studies that evaluated long-term follow-up in patients receiving LCIG. These data include analysis sets collected from four multicenter trials and a global long-term, multinational, observational registry study on efficacy and safety of LCIG in patients with advanced PD in routine care (GLORIA). These studies provide the largest dataset with long-term follow-up of LCIG in patients with advanced PD to date. The dataset was stratified to provide insights into patient populations that require high-dose levodopa [8–12].

## 2. Methods

### 2.1. Participants

#### 2.1.1. Phase III Program

Patients were eligible for inclusion if they had a diagnosis of idiopathic PD with severe motor fluctuations (≥3 hours of “Off” time per day) not adequately controlled by optimized PD therapy, were levodopa responsive, and were ≥30 years of age. Exclusion criteria for patients included any clinically significant medical, laboratory, psychiatric, or surgical issues as determined by the investigator to likely to interfere with participation in the study.

#### 2.1.2. GLORIA Registry

Patients were eligible for inclusion in the GLORIA registry if they exhibited severe motor fluctuations not adequately controlled by optimized PD therapy, were levodopa responsive, had ≤12 months of previous treatment with LCIG, and met additional eligibility criteria for LCIG treatment according to the European Commission Summary Product Characteristics and national reimbursement criteria, where applicable. Patients were also required to demonstrate a positive clinical response to LCIG administered via a temporary nasojejunal (NJ) tube prior to receiving a permanent PEG-J tube. Patients were enrolled from 75 movement disorder centers in Australia, Austria, Belgium, Bulgaria, Czech Republic, Denmark, France, Germany, Greece, Ireland, Italy, Netherlands, Norway, Romania, Slovenia, Spain, Switzerland, and the United Kingdom [13, 14].

### 2.2. Study Design and Treatment

#### 2.2.1. Phase III Program

Data were collected from patients with advanced PD who participated in a 12-week, randomized, double-blind, active-controlled parallel-group study [9] (NCT00660387/NCT0357994) and either the subsequent 52-week open-label extension study [12] (NCT00360568) or a separate 54-week open-label study [10] (NCT00335153). Patients were then eligible to enroll in an ongoing open-label, continued-access multinational extension study [11] (NCT00660673) where they could continue treatment until the product was available locally. The enrollment period was from November 2009 through October 2012. Further documented methods for each study can be found in the corresponding publications [9–12].

LCIG was continuously administered via a portable pump for 16 hours/day through PEG-J tubing inserted directly into the jejunum. For data collected from patients in the 12-week, randomized, double-blind, parallel-group study [9], the initial dose was determined based on the patients' previous day's oral levodopa dose. The dose was titrated for 4 weeks and then held stable for 8 weeks. When patients entered the extension study [12], the investigator determined whether to adjust the dose and/or taper or add adjunctive PD medications. For the open-label study [10], the dose was calculated on the previous day's oral levodopa dose. Additionally, all other PD medications were tapered before titration; after week 4, the investigator made the judgment as to whether the oral medications should again be added. In the continuation-of-access study [11], patients' initial LCIG doses were the same as those received at the end of the previous open-label LCIG study. Dose adjustments were made by the investigator as clinically indicated. Patients were allowed to self-administer additional LCIG doses to address the immediate need for symptom relief (e.g., motor function deterioration). Patients could also administer oral levodopa/carbidopa for supplemental bedtime or overnight doses [11].

#### 2.2.2. GLORIA Registry

GLORIA was a 24-month, multinational, noninterventional, observational registry of patients with advanced PD treated with LCIG in routine care. Details of the GLORIA registry methods have been published elsewhere [15].

LCIG treatment was initiated via a temporary NJ tube to verify drug efficacy and optimize the dose before being administered through PEG-J (according to local label and reimbursement criteria). Concomitant medications were permitted at the discretion of the treating physician.

All studies were conducted in accord with the Good Clinical Practice guideline as defined by the International Council on Harmonization, the Declaration of Helsinki, and all applicable federal and local regulations and institutional review boards [9–12, 15].

### 2.3. Safety Assessments

#### 2.3.1. Phase III Program

AEs in the phase III program were summarized for all patients who received open-label LCIG treatment (*N* = 412) through October 2016. AEs were coded using the *Medical Dictionary for Regulatory Activities* (MedDRA) and were tabulated by the MedDRA preferred term. Throughout the program, each event could be coded to more than one preferred term descriptive of the event. AEs presented are all treatment emergent and are included, regardless of causality. Procedure- and device-associated AEs were defined by an AbbVie-MedDRA preferred-term search strategy based on a medical review of MedDRA preferred terms to identify those that were potentially related to the procedure or long-term use of the device; these AEs were not included in this analysis as they are not relevant to the evaluation of the LCIG dose.

#### 2.3.2. GLORIA Registry

Adverse drug reactions (ADRs), defined as AEs that were considered by the investigator to have at least a reasonable possibility of having a causal relationship to LCIG treatment or the device delivery system, were recorded for the total duration of the registry and an additional 28 days following the last reported study date for each patient. ADRs were coded using MedDRA and classified by a potential relationship to the LCIG treatment and severity. Serious ADRs and complaints were monitored and recorded.

### 2.4. Efficacy Assessments

#### 2.4.1. Phase III Program

Efficacy outcomes were derived from a PD symptom diary recorded by patients and included the mean change from baseline to the last study visit in “Off” time, “On” time without TSD, and “On” time with TSD. The efficacy dataset included patients enrolled in the 54-week open-label phase III study (NCT00360568), which constituted the majority of the patients in the registration trials.

#### 2.4.2. GLORIA Registry

Efficacy outcomes included the mean change from baseline to the last study visit in Unified Parkinson's Disease Rating Scale (UPDRS) part IV items 39 (“Off ” time) and 32 (time with dyskinesia). Items 39 and 32 were modified using the rating instructions for corresponding parts 4.3 and 4.1 of the Movement Disorder Society (MDS)-UPDRS to allow for calculation of actual hours of “Off” time and “On” time with dyskinesias. MDS-UPDRS assessments were conducted in the “On” state.

### 2.5. Statistical Analysis

#### 2.5.1. Dosing Groups

In this post hoc analysis, patients enrolled in the phase III clinical trials were categorized into groups based on their mean total daily levodopa dose, <2000 mg or ≥2000 mg. For the GLORIA registry study, patients with ≥100 mL (2000 mg) daily levodopa dose at three or more study visits were placed into the ≥2000 mg dosage group. Results in high- and low-dosage groups were reported using descriptive statistics. The nonrandomized nature of the groups precluded any further comparative statistical analyses.

## 3. Results

### 3.1. Patients

Data were collected from a total of 412 patients in the phase III program and 356 patients in the GLORIA registry. Baseline patient characteristics are summarized for both studies in Tables 1 and 2. Patients from the phase III program and the GLORIA registry were similar in age (phase III program, 64.1 years; GLORIA registry, 66.6 years) and disease duration (phase III program, 12.3 years; GLORIA registry, 12.8 years) and were predominantly white (phase III program, 93%; GLORIA registry, 98%).

In the phase III program, a total of 72 (17%) patients required ≥2000 mg/day levodopa and 340 (83%) patients required <2000 mg/day. In the GLORIA registry, 47 (13%) patients required ≥2000 mg/day levodopa and 309 (87%) patients required <2000 mg/day to adequately treat motor symptoms. In both the phase III program and the GLORIA registry, more men than women required the ≥2000 mg/day dosage (phase III program, 22% of men vs 11% of women; GLORIA registry, 16.1% of men vs 9% of women). Patients in the ≥2000 mg/day levodopa groups generally had a higher body weight and higher baseline oral levodopa dose prior to initiating LCIG compared with the <2000 mg/day levodopa groups. Baseline demographics and disease severity (as measured by the PD diary and UPDRS) were generally comparable between dosage groups. In the GLORIA registry, patients in the ≥2000 mg group had a lower baseline dyskinesia duration than patients receiving <2000 mg/day levodopa.

### 3.2. Safety

Across dosage groups, the mean (standard deviation) study drug exposure was 1090.7 (872.6) days in the phase III program and 640.7 (198.3) days in the GLORIA registry.

The overall frequency of AEs and ADRs was higher in patients who required ≥2000 mg levodopa compared with patients requiring <2000 mg levodopa, although this difference was not tested with formal statistical analysis (Table 3). The AE/ADR profile includes events commonly associated with underlying PD, levodopa exposure, or the age group studied. In the phase III program, specific AEs that were observed at >10% higher frequency in the ≥2000 mg levodopa dosage group included fall, constipation, reemergence of Parkinson's disease symptoms, increased blood homocysteine, decreased weight, anxiety, vomiting, and arthralgia. In the GLORIA registry, ADRs that were observed at >3% of patients in the ≥2000 mg levodopa dosage group included decreased weight, polyneuropathy, and hallucination.

The overall frequency of serious AEs (SAEs) in the phase III program, excluding procedure- and device-associated AEs, was higher in patients who required ≥2000 mg than patients who required <2000 mg levodopa (63.9% vs 43.8%, respectively), although this difference was not tested with formal statistical analysis. This pattern was also observed for patients in the GLORIA registry (38.3% vs 28.8%, respectively; Table 4). Incidences of reemergence Parkinsonism (or parkinsonian symptoms), pneumonia, fall, and hip fracture were reported most frequently. Incidences of AEs and ADRs of special interest are listed in Table 5. In both datasets, dizziness, hallucination, and polyneuropathy occurred more frequently in patients who required ≥2000 mg dosage than in patients in the low-dosage group.

Overall, 18.7% of patients discontinued treatment due to an AE/ADR in the phase III program; 6.7% of patients discontinued in the GLORIA registry. Discontinuations in the phase III program due to nonprocedure- or device-associated AEs were slightly higher in the ≥2000 mg/day group vs the <2000 mg/day levodopa group (25% vs 17%, respectively). In the GLORIA registry, discontinuations due to ADRs were markedly lower among patients who received ≥2000 mg/day levodopa (0% of 47 patients) compared with patients who received <2000 mg/day (7.8%; Table 6).

### 3.3. Efficacy

Although statistical comparisons were not performed, descriptive statistics indicate that LCIG-treated patients from both dosage groups in the phase III program and GLORIA registry showed similar reductions from baseline in “Off” time and “On” time without TSD, as well as measurements of activities of daily living and quality of life (Table 7). In the phase III program (specifically, the 52-week open-label study), patients who required ≥2000 mg/day had a reduction from baseline in “Off” time of 4.3 hours compared with 4.5 hours in the <2000 mg/day levodopa group. Dyskinesia (“On” time with troublesome dyskinesia) was also reduced from baseline in the ≥2000 mg/day levodopa group by 0.2 hours and the <2000 mg/day levodopa group by 0.4 hours. UPDRS part II scores (activities of daily living) and Parkinson's Disease Questionnaire-39 (PDQ-39) summary index were similarly reduced from baseline in both dosage groups.

In the GLORIA registry, patients who required ≥2000 mg/day had a reduction from baseline in “Off” time of 4.9 hours compared with 3.8 hours in the <2000 mg/day levodopa group. In addition, dyskinesias (“On” time with dyskinesia) were likewise reduced from baseline in the ≥2000 mg/day levodopa group by 1.3 hours and in the <2000 mg/day levodopa group by 1.1 hours. UPDRS part II scores were reduced from baseline in both dosage groups, although the reduction was greater in the ≥2000 mg/day levodopa group. PDQ-8 summary index scores were reduced from baseline in both dosage groups, although to a somewhat greater degree in the <2000 mg/day levodopa group.

## 4. Discussion

This report expands on the long-term safety and efficacy of LCIG use in patients with advanced PD who participated in a pooled dataset of phase III clinical trials or in the GLORIA registry. These analyses intended to focus on details concerning a subset of the patient population who required levodopa dosages in excess of 2000 mg/day. Accordingly, data from the combined studies were stratified into two groups: those who required <2000 mg/day levodopa and those who required ≥2000 mg/day levodopa.

Patient baseline characteristics were generally similar between dosage groups, with a higher proportion of male patients in the high-dosage group. A retrospective chart review of more than 30,000 patients in Sweden found that the mean oral levodopa dose per day prescribed for men was significantly higher than the dose for women [16]. An assessment of 672 patients who received higher dosages (≥1200 mg/day) of oral levodopa revealed that 67% were men, and of 91 patients who received ≥2000 mg/day levodopa, 75% were men [16]. Nyholm et al. report that 26 of 44 patients with duodenal levodopa infusion required ≥1200 mg levodopa daily. Of these 26 patients, 21 (81%) were receiving levodopa monotherapy, with the median levodopa dosage at the last patient visit being 1990 mg/day. In this study, males made up 77% of the high-dosage group and 56% of the low-dosage group [16].

On average, patients who required ≥2000 mg/day levodopa had higher body weight and baseline oral levodopa doses compared with patients who received <2000 mg/day levodopa. Pharmacologically, it is not surprising that higher doses were required in individuals with higher body mass indexes. Results from pharmacokinetic studies show that higher body weight is associated with lower plasma levels of levodopa [17]. Findings from previous studies show that women exhibit a higher levodopa bioavailability compared with men when factoring weight-corrected area under the concentration-time curve (AUC) [18]. Women generally show lower body weight [19], which may contribute to the differences seen in levodopa requirements between male and female patients. Finally, it is also likely that differences in the delivered dose reflect intraindividual variability in intestinal absorption, liver metabolism, and transport (both jejunal and blood-brain barrier) [20, 21].

The reported rate of AEs and ADRs was higher in patients requiring ≥2000 mg/day levodopa compared with patients with lower levodopa treatment dosages but was still consistent with the established safety and tolerability profile of LCIG [8, 10, 12]. Specifically, our findings suggest a higher incidence of weight loss and the reemergence of PD symptoms in patients who received ≥2000 mg/day levodopa compared with patients who received <2000 mg/day levodopa. Oral levodopa may be associated with a dose-dependent effect on weight loss in PD patients [22]. This finding may be due to more advanced stages of PD as a contributor to weight loss (and the reemergence of PD symptoms) because dose escalation is common as the disease progresses. Results from the study by Akbar et al. show an association between weight loss in PD patients and increased disease severity, older age, more comorbidities, and higher rates of levodopa usage [23]. Fabbri et al. observed a direct correlation between weight loss and the percent of the day spent with dyskinesia when on LCIG therapy; they also found a correlation between nutritional status (as measured by the Mini Nutritional Assessment) and levodopa equivalent daily dose [24]. Weight loss in PD is likely a multifaceted phenomenon that occurs subsequent to many contributing factors, including underlying pathological changes (e.g., metabolic and gastrointestinal dysfunction), ageing, and administration of levodopa.

We observed higher frequency of polyneuropathy and polyneuropathy-related AEs in patients receiving ≥2000 mg/day levodopa compared with patients who received <2000 mg/day, although we did not perform statistical analyses to confirm this difference. It should be noted that, in the GLORIA registry, there was not a structured clinical, neurophysiological, or laboratory follow-up of patients regarding vitamin B12 and homocysteine levels, so these data should not be overinterpreted. A study that compared LCIG (mean levodopa dosage, 1909 mg/day) with oral levodopa (mean dosage, 1047 mg/day) found a similar incidence of axonal neuropathy in both treatment groups, although neurographic abnormalities were more severe in patients treated with LCIG [25]. Some studies have suggested that the frequency of polyneuropathy is more likely to be associated with the duration of the cumulative exposure than with peak doses during LCIG therapy [26, 27]. Other studies have indicated a link between polyneuropathy and a higher levodopa equivalent daily dose [28]. However, other factors may be linked to levodopa-related polyneuropathy, including a disruption in the metabolic breakdown of levodopa, causing low levels of vitamin B12, vitamin B6, and folate [28, 29] and high homocysteine and methylmalonic acid [28–30] and/or BMI reduction [30]. Levodopa-related polyneuropathy is evidently a complex phenomenon that requires careful monitoring of neuropathic symptoms and vitamin deficiencies, particularly when higher levodopa doses are used.

We observed a higher frequency of hallucination in the ≥2000 mg/day levodopa group compared with patients in the <2000 mg/day levodopa group in both the phase III program and the GLORIA registry. Hallucinations are a common occurrence in advanced PD and have been linked with the duration of treatment and total daily levodopa dose, as well as with patient age and cognitive status [31–33]. Prospective, long-term studies did not find associations between hallucinations and high doses of levodopa or treatment duration [31, 34]. In our analysis, hallucinations were more common in patients with higher levodopa doses, despite similar baseline disease characteristics. However, it is also possible that this observation of increased prevalence was related to a more severe disease requiring higher doses of levodopa for motor control. Furthermore, hallucinations could also be related to other antiparkinsonian medications (e.g., dopamine agonists).

In the GLORIA registry, patients in the ≥2000 mg/day levodopa group had a lower baseline for the duration of dyskinesia than did patients in the <2000 mg/day levodopa group, suggesting that these patients may be less susceptible to dyskinesia [16]. These findings suggest that patients with a lower susceptibility to dyskinesia can be titrated to higher LCIG doses to achieve similar motor control. However, observation of a broad range of LCIG doses may reflect not only differences in levodopa intestinal absorption but also differences in pharmacodynamic response to dopamine, including control and susceptibility to dyskinesias.

Improvements in “Off” time were similar between dosage groups. Due to the progressive nature of PD, dose escalation is necessary to continue providing the most optimized treatment plans for each patient. Thus, it is not surprising that higher doses of levodopa are effective in providing relief from PD-related motor impairment. While speculative, it is possible that, with a more simplistic regimen found in LCIG monotherapy, patient adherence to treatment was more conducive to improved PD symptoms. In addition to improved motor symptoms, the ability to perform activities of daily living, as well as patients' quality of life, was equally improved in both dosage groups. The efficacy in routine clinical practice as demonstrated by the GLORIA registry data further confirms the positive findings from the placebo-controlled phase III program.

Discontinuations because of AEs were similar between dosage groups in the phase III program. In the GLORIA registry, discontinuations due to ADRs were lower in patients requiring ≥2000 mg/day levodopa compared with those receiving <2000 mg/day. A relatively low rate of discontinuations in the GLORIA registry study suggests continued patient compliance outside a structured trial setting and satisfaction with the overall treatment.

The reported study findings are limited because of the differences between study design, the post hoc nature of the analyses, patient selection, and recruitment among the trials. Study design specifics that may impact the overall perceived benefit of LCIG include differences in patients who initiated LCIG in an open-label vs a double-blind setting. Patients were recruited from movement disorder centers where patient care is more specialized to advanced PD and management protocols likely differ from those of traditional hospital settings. Furthermore, the nonrandomized nature of the 2 groups precluded any statistical analyses other than descriptive statistics. Other types of analyses or other studies are needed to determine if there is a statistical difference between these dosing groups for safety and efficacy/effectiveness parameters. Despite these limitations, we provide here the largest prospective dataset to date that evaluated the safety and efficacy of LCIG, with a focus on patients who required ≥2000 mg/day levodopa.

In summary, we provide further data on the safety and efficacy profile of high-dosage (≥2000 mg/day) levodopa through LCIG administration in patients with advanced PD. These findings inform the use of high doses of LCIG in the long term, with a similar safety and efficacy profile as previously established and observed in lower dosing regimens. Of note, a higher number of AEs were observed in the higher dosage group, but this number was within what is known and accepted for LCIG. These data will aid clinicians in appropriate management and best-care practices for patients who require higher doses of LCIG for adequate control of motor fluctuations.

## Figures and Tables

**Table 1:** Phase III program baseline patient demographics and clinical characteristics.

Characteristics	Mean (SD)^a^		
	Levodopa <2000 mg/day (*n* = 340)	Levodopa ≥2000 mg/day (*n* = 72)	Overall (*N* = 412)
Age, years	64.1 (9.1)	64.0 (8.4)	64.1 (8.9)
Sex, *n* (%)			
Female	151 (44)	18 (25)	169 (41)
Male	189 (56)	54 (75)	243 (59)
Race, *n* (%)			
White	312 (92)	69 (96)	381 (93)
Asian	23 (7)	3 (4)	26 (6)
Black/African American	4 (1)	—	4 (1)
American Indian or Alaska Native	1 (0.3)	—	1 (0.2)
BMI, kg/m^2^	24.7 (4.5)^b^	25.5 (5.3)^c^	24.8 (4.7)^d^
Weight, kg	70.2 (15.6)^e^	75.9 (18.7)	71.2 (16.3)^f^
PD duration, years	12.3 (5.6)	12.0 (5.1)	12.3 (5.5)
Baseline oral levodopa dosage, mg/d	1000.3 (499.6)	1464.8 (693.3)	1080.7 (565.3)
UPDRS part II score	16.4 (7.1)^g^	16.7 (7.5)^h^	16.4 (7.2)^i^
UPDRS part III score	27.5 (13.7)^j^	27.5 (14.5)^h^	27.5 (13.9)^k^
Normalized “Off” time, hours	6.7 (2.3)^l^	6.7 (2.2)	6.7 (2.3)^m^
Normalized “On” time without TSD, hours	7.7 (2.5)^l^	8.1 (2.0)	7.8 (2.4)^m^
Normalized “On” time with TSD, hours	1.6 (2.0)^l^	1.2 (1.8)	1.5 (2.0)^m^
Previous PD treatment, *n* (%)			
Oral levodopa	340 (100)	71 (99)^n^	411 (100)^n^
Dopamine agonists	207 (61)	49 (68)	256 (62)
COMT inhibitors	101 (30)	30 (42)	131 (32)
MAO-B inhibitors	68 (20)	11 (15)	79 (19)
Amantadine	120 (35)	17 (24)	137 (33)

“On”/“Off” time was normalized to a 16-hour waking day and averaged for the three days prior to each study visit. ^a^Data are shown as mean (SD) except where indicated as *n* (%). ^b^*n*: 336; ^c^*n*: 71; ^d^*n*: 407; ^e^*n*: 338; ^f^*n*: 410; ^g^*n*: 315; ^h^*n*: 64; ^i^*n*: 379; ^j^*n*: 313; ^k^*n*: 377; ^l^*n*: 334; ^m^*n*: 406; ^n^data collection issue for one patient. BMI: body mass index; COMT: catechol-O-methyl transferase; MAO-B: monoamine oxidase B; PD: Parkinson's disease; SD: standard deviation; TSD: troublesome dyskinesia; UPDRS: Unified Parkinson's Disease Rating Scale.

**Table 2:** GLORIA registry baseline patient demographics and clinical characteristics.

Characteristics	Mean (SD)^a^		
	Levodopa <2000 mg/day (*n* = 309)	Levodopa ≥2000 mg/day (*n* = 47)	Overall (*N* = 356)
Age, years	66.6 (8.4)	66.6 (8.4)	66.6 (8.4)
Sex, *n* (%)			
Female	132 (43)	13 (28)	145 (41)
Male	177 (57)	34 (72)	211 (59)
Race, *n* (%)			
White	301 (99)	43 (94)	344 (98)
Asian	2 (0.7)	2 (4.3)	4 (1.1)
Black/African American	1 (0.3)	—	1 (0.3)
American Indian/Alaska Native	1 (0.3)	1 (2.2)	2 (0.6)
BMI, kg/m^2^	25.0 (4.0)^b^	26.1 (6.2)^c^	25.1 (4.4)^d^
Weight, kg	70.5 (13.8)^e^	73.7 (17.7)^f^	70.9 (14.4)^g^
PD duration, years	13.0 (6.5)^h^	12.0 (4.7)^i^	12.8 (6.3)^j^
Baseline oral levodopa dosage, mg/d	876.9 (443.3)^k^	1156.3 (418.3)^l^	913.6 (449.5)^m^
UPDRS part II score	16.1 (9.8)^n^	17.8 (9.7)^o^	16.3 (9.8)^p^
UPDRS part III score	25.0 (12.2)^q^	21.8 (10.2)^r^	24.6 (12.0)^s^
Modified UPDRS part IV item 39 “Off” time, hours	5.9 (3.0)^t^	6.8 (3.9)^u^	6.0 (3.2)^v^
Modified UPDRS part IV item 32 “On” time with TSD, hours	4.5 (3.8)^w^	3.3 (3.7)^x^	4.3 (3.8)^y^
Previous PD treatment, *n* (%)			
Oral levodopa	269 (87)	41 (87)	310 (87)
Dopamine agonists	256 (83)	43 (92)	299 (84)
COMT inhibitors	204 (66)	39 (83)	243 (68)
MAO-B inhibitors	154 (50)	25 (53)	179 (50)
Amantadine	130 (42)	18 (38)	148 (42)
Others	48 (16)	10 (21)	58 (16)

^a^Data are shown as mean (SD) except where indicated as *n* (%). ^b^*n*: 219; ^c^*n*: 34; ^d^*n*: 253; ^e^*n*: 245; ^f^*n*: 38; ^g^*n*: 283; ^h^*n*: 281; ^i^*n*: 47; ^j^*n*: 328; ^k^*n*: 304; ^l^*n*: 46; ^m^*n*: 350; ^n^*n*: 200; ^o^*n*: 30; ^p^*n*: 230; ^q^*n*: 224; ^r^*n*: 34; ^s^*n*: 258; ^t^*n*: 182; ^u^*n*: 29; ^v^*n*: 211; ^w^*n*: 187; ^x^*n*: 28; ^y^*n*: 217. BMI: body mass index; COMT: catechol-O-methyl transferase; MAO-B: monoamine oxidase; PD: Parkinson's disease; SD: standard deviation; TSD: troublesome dyskinesia; UPDRS: Unified Parkinson's Disease Rating Scale.

**Table 3:** AE and ADR summary.

Phase III program	Patients, *n* (%)		
	Levodopa <2000 mg/day (*n* = 340)	Levodopa ≥2000 mg/day (*n* = 72)	Overall (*N* = 412)
Patients with any AE^a^	315 (92.6)	71 (98.6)	386 (93.7)
Select AEs and AEs occurring in ≥10% of patients overall			
Fall	76 (22.4)	27 (37.5)	103 (25.0)
Insomnia	83 (24.4)	17 (23.6)	100 (24.3)
Nausea	67 (19.7)	21 (29.2)	88 (21.4)
Constipation	64 (18.8)	22 (30.6)	86 (20.9)
Decreased vitamin B6	71 (20.9)	15 (20.8)	86 (20.9)
Urinary tract infection	69 (20.3)	16 (22.2)	85 (20.6)
*Parkinson's disease*^b^	48 (14.1)	22 (30.6)	70 (17.0)
*Increased blood homocysteine*	47 (13.8)	19 (26.4)	66 (16.0)
Decreased weight	48 (14.1)	18 (25.0)	66 (16.0)
Anxiety	49 (14.4)	17 (23.6)	66 (16.0)
Dyskinesia	49 (14.4)	14 (19.4)	63 (15.3)
Depression	52 (15.3)	11 (15.3)	63 (15.3)
Back pain	42 (12.4)	8 (11.1)	50 (12.1)
Orthostatic hypotension	39 (11.5)	10 (13.9)	49 (11.9)
*Vomiting*	29 (8.5)	16 (22.2)	45 (10.9)
Diarrhea	35 (10.3)	9 (12.5)	44 (10.7)
Headache	34 (10.0)	9 (12.5)	43 (10.4)
*Arthralgia*	28 (8.2)	13 (18.1)	41 (10.0)
Dizziness	18 (5.3)	10 (13.9)	28 (6.8)
Sedation	1 (0.3)	—	1 (0.2)

GLORIA registry	Levodopa <2000 mg/day (*n* = 309)	Levodopa ≥2000 mg/day (*n* = 47)	Overall (*N* = 356)

Patients with any ADR^c^	153 (49.5)	38 (80.9)	191 (53.7)
ADRs occurring in ≥3% of patients overall^a^			
*Decreased weight*	16 (5.2)	8 (17.0)	24 (6.7)
*Polyneuropathy*	12 (3.9)	4 (8.5)	16 (4.5)
*Hallucination*	9 (2.9)	3 (6.4)	12 (3.4)

AEs and ADRs in italics represent events reported in the ≥2000 mg dose group at twice the rate of that reported in the low-dose group. ^a^Excluding those associated with the procedure/device. ^b^Refers to the reemergence of Parkinson's disease symptoms, often due to a problem with drug delivery. ^c^ADRs were AEs deemed by the investigator to have at least a reasonable possibility of a causal relationship to the treatment drug/device. ADR: adverse drug reaction; AE: adverse event.

**Table 4:** Summary of serious AEs and serious ADRs.

Phase III program	Patients, *n* (%)		
	Levodopa <2000 mg/day (*n* = 340)	Levodopa ≥2000 mg/day (*n* = 72)	Overall (*N* = 412)
Patients with any serious AE	149 (43.8)	46 (63.9)	195 (47.3)
Any SAE occurring in ≥2% of patients overall			
Pneumonia	21 (6.2)	5 (6.9)	26 (6.3)
*Parkinson's disease*^a^	10 (2.9)	5 (6.9)	26 (6.3)
Fall	10 (2.9)	4 (5.6)	14 (3.4)
Death	10 (2.9)	1 (1.4)	11 (2.7)
*Hip fracture*	7 (2.1)	4 (5.6)	11 (2.7)
*Pneumonia aspiration*	7 (2.1)	3 (4.2)	10 (2.4)
*Polyneuropathy*	6 (1.8)	3 (4.2)	9 (2.2)

GLORIA registry	Levodopa <2000 mg/day (*n* = 309)	Levodopa ≥2000 mg/day (*n* = 47)	Overall (*N* = 356)

Patients with any serious ADR^b^	89 (28.8)	18 (38.3)	107 (30.1)
Any serious ADRs occurring in ≥2% of patients overall^c^			
Parkinsonism^a^	7 (2.3)	—	7 (2.0)
Parkinson's disease^a^	6 (1.9)	1 (2.1)	7 (2.0)

AEs and ADRs in italics represent events reported in the ≥2000 mg dose group at twice the rate of that in the low-dose group. ^a^Refers to the reemergence of Parkinson's disease symptoms, often due to a problem with drug delivery. ^b^ADRs were AEs deemed by the investigator to have at least a reasonable possibility of a causal relationship to the treatment drug/device. ^c^Excluding those associated with the procedure/device. ADR: adverse drug reaction; AE: adverse event; SAE: serious adverse event.

**Table 5:** Patients with select AEs and ADRs of special interest.

Phase III program	Patients, *n* (%)		
	Levodopa <2000 mg/day (*n* = 340)	Levodopa ≥2000 mg/day (*n* = 72)	Overall (*N* = 412)
Abuse liability AEs			
Intentional overdose	1 (0.3)	—	1 (0.2)
Psychomotor hyperactivity	—	1 (1.4)	1 (0.2)
Sleep/sleep attack-related AEs			
Sleep attacks	30 (8.8)	9 (12.5)	39 (9.5)
Somnolence	8 (2.4)	4 (5.6)	12 (2.9)
Hallucination/psychosis-related AEs			
Hallucination	25 (7.4)	13 (18.1)	38 (9.2)
Hallucination, auditory	4 (1.2)	—	4 (1.0)
Hallucination, tactile	2 (0.6)	—	2 (0.5)
Hallucination, visual	11 (3.2)	1 (1.4)	12 (2.9)
Hallucination, mixed	1 (0.3)	—	1 (0.2)
Psychotic disorder	3 (0.9)	3 (4.2)	6 (1.5)
Polyneuropathy-related AEs^a^	32 (9.4)	17 (23.6)	49 (11.9)
Polyneuropathy-related AEs reported in >1% of patients overall^b^			
Polyneuropathy	15 (4.4)	10 (13.9)	25 (6.1)
Peripheral neuropathy	5 (1.5)	1 (1.4)	6 (1.5)
Peripheral sensory neuropathy	5 (1.5)	1 (1.4)	6 (1.5)

GLORIA registry	Levodopa <2000 mg/day (*n* = 309)	Levodopa ≥2000 mg/day (*n* = 47)	Overall (*N* = 356)

Somnolence	2 (0.6)	1 (2.1)	3 (0.8)
Hallucination/psychosis-related ADRs^b^			
Hallucination	9 (2.9)	3 (6.4)	12 (3.4)
Hallucination, visual	1 (0.3)	1 (2.1)	2 (0.6)
Psychotic disorder	6 (1.9)	1 (2.1)	7 (2.0)
Polyneuropathy-related ADRs^b^ reported in >1% of patients overall			
Polyneuropathy	12 (3.9)	4 (8.5)	16 (4.5)
Peripheral neuropathy	4 (1.3)	1 (2.1)	5 (1.4)
Peripheral sensory neuropathy	1 (0.3)	3 (6.4)	4 (1.1)

^a^Based on the Standard MedDRA Query narrow search of Guillain–Barré syndrome and peripheral neuropathy. ^b^Polyneuropathy AEs not listed in the table include Guillain–Barré syndrome, which occurred in two patients (*n*/*N* = 2/72, 2.8%) who required ≥2000 mg dose for PD symptom control. ^b^ADRs were AEs deemed by the investigator to have at least a reasonable possibility of a causal relationship to the treatment drug/device. ADR: adverse drug reaction; AE: adverse event; PD: Parkinson's disease.

**Table 6:** AEs and ADRs that led to discontinuation.

Phase III program	Patients, *n* (%)		
	Levodopa <2000 mg/day (*n* = 340)	Levodopa ≥2000 mg/day (*n* = 72)	Overall (*N* = 412)
Incidence of AEs leading to discontinuation	59 (17.4)	18 (25.0)	77 (18.7)
AEs leading to discontinuation in >2 patients overall			
Death	9 (2.6)	1 (1.4)	10 (2.4)
Pneumonia	4 (1.2)	1 (1.4)	5 (1.2)
Myocardial infarction	3 (0.9)	1 (1.4)	4 (1.0)
Cardiac arrest	1 (0.3)	2 (2.8)	3 (0.7)
Fall	2 (0.6)	1 (1.4)	3 (0.7)
Parkinson's disease^a^	3 (0.9)	—	3 (0.7)

GLORIA registry	Levodopa <2000 mg/day (*n* = 309)	Levodopa ≥2000 mg/day (*n* = 47)	Overall (*N* = 356)

Patients with ≥1 ADR^b^ leading to discontinuation	24 (7.8)	—	24 (6.7)
ADRs leading to discontinuation in 2 patients^c^ overall	^*∗*^No ADRs met this criterion		

^a^Refers to the reemergence of Parkinson's disease symptoms, often due to a problem with drug delivery. ^b^ADRs were AEs deemed by the investigator to have at least a reasonable possibility of a causal relationship to the treatment drug/device. ^c^Excluding those associated with the procedure/device. ADR: adverse drug reaction; AE: adverse event.

**Table 7:** Select efficacy outcomes.

Phase III program^a^	Mean (SD) change from baseline to the last visit	
	Levodopa <2000 mg/day (*n* = 253^b^)	Levodopa ≥2000 mg/day (*n* = 54^b^)
“Off” time, hours	–4.5 (2.8)	–4.3 (3.1)
“On” time without TSD, hours	4.9 (3.4)	4.5 (3.3)
“On” time with TSD, hours	–0.4 (2.8)	–0.2 (2.8)
UPDRS part II score	–4.6 (6.3)	–3.5 (7.0)
PDQ-39 summary index	–7.0 (13.4)	–6.7 (17.4)

GLORIA registry	Levodopa <2000 mg/day (*n* = 178^b^)	Levodopa ≥2000 mg/day (*n* = 29^b^)

Modified UPDRS part IV item 39: “Off” time, h	–3.8 (3.4)	–4.9 (4.2)
Modified UPDRS part IV item 32: “On” time with dyskinesia, h	–1.1 (4.7)	–1.3 (4.5)
UPDRS part II score	–0.9 (9.2)	–3.0 (8.2)
PDQ-8 summary index	–7.4 (20.0)	–5.2 (25.7)

“On”/“Off” time was normalized to a 16-hour waking day and averaged for the three days prior to each study visit. ^a^Efficacy analyses include only data from patients enrolled in the 12-month open-label phase III study (study II); baseline values for this population were similar to those presented for the phase III program population in Table 1. ^b^Number of patients at the last visit. PDQ-8: Parkinson's Disease Questionnaire-8; SD: standard deviation; TSD: troublesome dyskinesia; UPDRS: Unified Parkinson's Disease Rating Scale.

## Data Availability

These clinical trial data can be requested by any qualified researchers who engage in rigorous, independent scientific research and will be provided following the review and approval of a research proposal and statistical analysis plan (SAP) and execution of a data sharing agreement (DSA). Data requests can be submitted at any time, and the data will be accessible for 12 months, with possible extensions considered. For more information on the process, or to submit a request, visit the following link: https://www.abbvie.com/our-science/clinicaltrials/clinical-trials-data-and-information-sharing/data-and-information-sharing-with-qualifiedresearchers.html.
